# Inhibitory effects of non-thermal atmospheric plasma on *Yersinia enterocolitica* and *Staphylococcus aureus* in the Korean traditional non-fermented *kimchi* “*Geotjeori*”

**DOI:** 10.1016/j.heliyon.2023.e19575

**Published:** 2023-09-02

**Authors:** So Hee Kim, Sung-Hee Park, Sung Gi Min, Shin Young Park

**Affiliations:** a Department of Seafood Science and Technology, Institute of Marine Industry, Gyeongsang National University, Tongyeong 53064, Republic of Korea; b Practical Technology Research Group, World Institute of Kimchi, Gwangju 61755, Republic of Korea

**Keywords:** Non-thermal atmospheric plasma, *Geotjeori*, *Yersinia enterocolitica*, *Staphylococcus aureus*, Quality

## Abstract

Food-borne bacteria have frequently been detected in *kimchi*, a representative and traditional fermented ethnic food of Korea. This study investigated the effect of atmospheric dielectric barrier discharge (DBD) plasma treatment (1.1 kV, 43 kHz, N_2_: 1.5 m/s, 5–60 min) on reduction of *Yersinia enterocolitica* and *Staphylococcus aureus* and on quality parameters in *Geotjeroi*, a non-fermented *kimchi*. A decrease of 0.12/0.09, 0.19/0.19, 0.34/0.45, 0.64/0.72, and 1.13/1.12 log_10_ CFU/g was observed by 5, 10, 20, 30, and 60 min of DBD plasma, respectively. D-value of 52.83 and 51.95 min was determined for *Y. enterocolitica* (R^2^ = 0.99) and *S. aureus* (R^2^ = 0.98) using the first order kinetics model. The quality parameters (pH, Brix, and hardness) were not significantly different (*P* > 0.05) between treated and untreated *Geotjeori*. Moreover, a decrease of >1 log_10_ CFU/g, for both bacteria was observed without any change in the quality of *Geotjeori*. These findings imply that DBD plasma treatment enhances *Geotjeori* safety and protects product from microbial risk.

## 1 Introduction

*Kimchi* is the one of the most popular traditional foods in Korea. It is usually made and fermented by mixing fresh main ingredients such as salted cabbage and radish, red pepper, garlic, ginger and fermented seafood (*Jeotgal*). There are more than 200 types of *kimchi*, depending on the region, type and characteristics of ingredients, and method of preparation [1]. It is prepared with cabbage, radish, cucumber, and green onion. Among them, cabbage and radish *kimchi* are the most popular in Korea. *Kimchi* contains various phytochemicals such as vitamins, minerals, organic acids, dietary fibers, and phenolic compounds [2]. Thus, it is known to exhibit various functions, such as antioxidant, and anticancer effects, and prevent hypertension [2]. *Kimchi* is a fermented food that is beneficial to health and contains lactic acid-producing bacteria. The optimum pH is 4.1–5.0. Lactic acid bacteria prevent the growth of pathogenic bacteria because of their antibacterial properties.

*Geotjeori kimchi*, a non-fermented *kimchi*, is an alternative for those who resist sourness, of fermented kimchi. *Geotjeori* is salted for some time and seasoned with cabbage. Unlike *kimchi*, it tastes shallow as it is pickled only on the outside. It can be consumed immediately after preparation or within 1–2 days after refrigeration. As the preparation is simple and short, people usually enjoy eating *Geotjeori* at home, food service facilities, and Korean restaurants. However, *Geotjeori kimchi* prepared without fermentation (nearly in a raw state) is more likely to be contaminated with pathogenic bacteria than fermented *kimchi*.

*Yersinia enterocolitica* is a common pathogen with a growth temperature range of 4–42 °C and an optimum temperature of 28–29 °C. It is a low-temperature bacterium that can grow at a refrigeration temperature of 5 °C and grows optimally at 25 °C but requires additional nutrients to grow at 37 °C [3]. Recently, it has attracted attention as a low-temperature intestinal pathogenic bacterium because of its ability to proliferate even at refrigeration temperatures [4]. It can cause food poisoning through consumption of refrigerated foods, such as cabbage or water. *Yersinia enterocolitica* is reported to have similar prevalence of bacterial enteritis as *Salmonella* in many countries worldwide [4]. The causative foods of *Y. enterocolitica* contamination include contaminated water, vegetables, dairy products, and undercooked meat products. Recently, *Y. enterocolitica* has also been detected in some *kimchi* (Anonymous, 2021).

*Staphylococcus aureus* can grow at 8–45 °C, with optimum growth at 35–38 °C [5]. It is widely distributed in nature and the most common cause of food poisoning worldwide; moreover, it can causes purulent diseases. As the bacteria is present on human skin, when a person with purulent disease comes in contact with food, the food is highly likely to be contaminated [6]. In a study on microbial contamination, *S. aureus* was detected in raw materials, cooking utensils, cooking staff's hands, and in the final cooked food [7]. A study has reported the presence of *S. aureus* in sushi, *gimbap* (food wrapped with seaweed and filled with rice and side dishes), meat products, cakes, and *naengmyeon* (Korean unique cold noodle dish) sold in the market [6]. In addition, since kimchi is generally made by hand, it can be contaminated with *S. aureus*, and in this regard, there is a report that *S. aureus* is able to survive as potential pathogens in *kimchi* [8].

Therefore, *Y. enterocolitica* and *S. aureus* are food poisoning bacteria that must be managed in *kimchi* such as *Geotgeori*. However, there is still a lack of studies on microbiological safety evaluation and microbial reduction of *Geotgeori* against these two bacteria.

In the food industry, heat treatment is used to produce safe food without contaminated by pathogenic microorganisms, but problems arise due to excessive heat treatment. Regarding these issues, alternative technologies such as high-pressure treatment and non-thermal plasma are being investigated for the inactivation of microbial contamination in food. Plasma, as quasi-neutral ionized gas, is the highly energized fourth state of substance containing ions, free electrons, atoms, and molecules in their fundamental and excited states with a net neutral charge [9]. Atmospheric plasma produces uniform plasma and it is not expensive and continuous processing is possible. Therefore, it can be applied to the agriculture and food industries at a low cost, and in recent years, non-thermal plasma is considered as an promising technology for removing microbiological contamination in food industry [10]. Atmospheric plasma includes methods such as dielectric barrier discharge (DBD), corona discharge, microwave discharge, and arc discharge [11]. In atmospheric plasma, DBD plasma refers to plasma discharged at a barrier between two parallel metal electrodes [12]. Since DBD plasma can discharge high-power without electrical impact, it is applied to biochemical fields such as cancer treatment, wound healing, and blood coagulation [11]. In addition, it can process large areas for the treatment target, and there is also a report that the shelf life of food is extended after DBD plasma exposure [11,13]. Also studies have specifically investigated the sterilization effect of DBD plasma against microorganisms such as molds [14], *Escherichia coli* and *Bacillus cereus* [15], human norovirus [16], and *S. aureus* [9]. Despite these advantages, research is systematically and extensively conducted in Korea and is not being conducted in depth. Therefore, further studies are needed to evaluate the sterilization effect of DBD plasma in various foods and against pathogenic microorganisms. In particular, no attempt has been made to evaluate the effectiveness of DBD plasma treatment for *Y. enterocolitica* and *S. aureus* of *Geotjeori*.

Therefore, in this study, we evaluated non-thermal atmospheric plasma sterilization effect against *Y. enterocolitica*, which is mainly detected in refrigerated foods, and *S. aureus* with a high prevalence of food poisoning using *Geotjeori*. In addition, pH, Brix value, and texture of *Geotjeori* were measured to ensure the overall quality of *Geotjeori* during evaluation of antibacterial effect of atmospheric plasma.

## 2 Materials and methods

### 2.1 Bacterial strain

*Yersinia enterocolitica* (ATCC 23,715) and *Staphylococcus aureus* (ATCC 6538, ATCC 12,600, and NCCP 20,220) stock cultures were stored at −80 °C in tryptic soy broth (TSB; Difco Laboratories, Detroit, MI, USA). Each strain was inoculated into BD™ TSB (Difco Laboratories). *Yersinia enterocolitica* was cultured at 30 °C and *Staphylococcus aureus* at 37 °C for 18−24 h. This process was repeated twice. The cultured strains were centrifuged at 5400×*g* for 10 min at 4 °C, and the precipitated pellets were suspended in of 9 mL 0.85% NaCl. This suspension was serially diluted using 0.85% NaCl.

### 2.2 Preparation of Geotjeori and bacterial inoculation

**Fig. 1:**
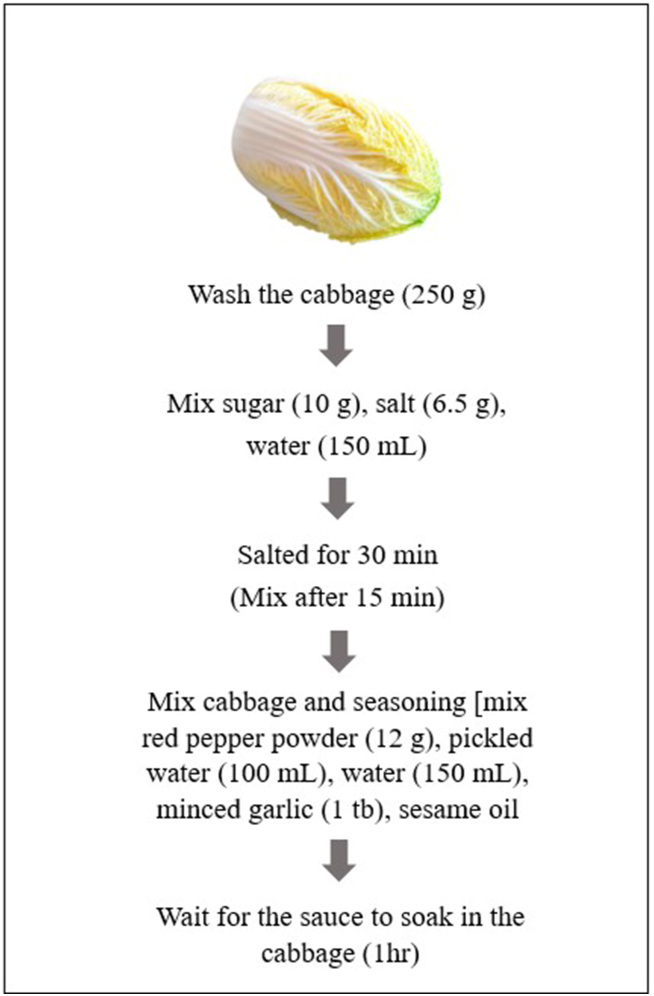
Manufacturing process of *Geotjeori* before dielectric barrier discharge (DBD) plasma treatment.

The ingredients and manufacturing process of *Geotjeori* is illustrated in Table 1 and Fig. 1, respectively. Cabbage, the main ingredient of *Geotjeori*, was purchased from plants grown in *Yeongwol-gun*, *Gangwon-do*. The red pepper powder under the product name of “chammas-cheonggyeol-gochusgalu” was used for seasoning.

**Table 1:** Seasonings used for *Geotjeori* preparation.

Materials	Amount^a^
Salt	6.5 g
Sugar	10 g
Water	150 mL
Red pepper	12 g
Minced garlic	1 tb
Sesame oil	2.5 g

a Standard of cabbage 250 g.

*Geotjeori* was cut into 3 g, 2.5 × 2.5 × 0.5 cm (L × W × T) pieces using sterilized scissors. Each of the two bacterial strains (100 μL) was inoculated into these pieces placed in sterilized Petri dish (90 × 15 mm). After inoculation, it was allowed to absorb in a biological safety cabinet (CHC Lab Co. Ltd., Daejeon, Korea) for 1 h. The final concentration of each bacterial strain was approximately 10^3^–10^4^ log CFU/g.

### 2.3 DBD plasma treatment

A (DBD) plasma device (μ‐DBD Surface Plasma Generator, Model; Micro DBD Plasma) was used in this experiment was supplied by the Plasma Biomedicine Institute (Plasma Bioscience Research Center, Seoul, Korea) [14]. The device was turned at least 10 min before the start of experiment. And the surface of *Geotjeori* inoculated with *Y. enterocolitica* and *S. aureus* were treated with DBD plasma exposure for 5, 10, 20, 30 and 60 min. Distance from the plasma-emitting electrode was maintained at 3 mm. A silver electrode (thickness: 10 μm) was attached to the glass plate (1.8 mm) using the screen-printing method. The dielectric material consisted of SiO_2_, which was screen printed at 100 μm in thickness. A metal mesh grid was attached to the back of the glass and used as a grounded electrode, which may induce gas flow to the mesh surface through the gas injection hole made using polylactic acid. DBD plasma was generated on the back surface between the glass and metal mesh grids at a nitrogen flow rate of 1.5 L/min. Under a driving frequency of 43 kHz, DBD plasma showed voltage current characteristics, with a discharge voltage of 1 kV and a discharge peak current of 40 mA. DBD plasma used in the experiment had a minimum discharge voltage of 1.1 kV required for plasma production. The optical emission spectra were obtained at an integration time of 10 s and a point 3 mm from the mesh surface using a spectrometer (HR 4000; Ocean Optics).

### 2.4 Modeling of microbial reduction

DBD plasma reduction of *Y. enterocolitica* and *S. aureus* in *Geotjeori* was modeled using the first-order kinetics, as follows:

$$\log\frac{N_{0}}{N}=\frac{k}{2.303}\times t$$

where N_0_ is the initial *Y. enterocolitica* and *S. aureus* titer (CFU), N is the *Y. enterocolitica* and *S. aureus* titer (CFU) at time t, t is the DBD exposure time (min), k is the reduction rate constant, and D-value is the decimal reduction time (min).

Hence, it can be characterized by a single rate constant “k” or its reciprocal, the D-value, which provides a quantitative measure of the resistance against an applied lethal agent and is calculated using the equation D = 2.303/k.

### 2.5 Measurement of pH and Brix value

For measuring pH, three samples and 27 g of distilled water were placed in sterilized bag and mixed using a stomacher (BagMixer; Interscience Inc., Troy, NY) for 1 min. The filtered liquid was measured using a pH meter (Orion Star A211; Thermo Fisher Scientific, MI, USA).

For measuring the Brix value, 3 g of *Geotjeori* and 3 g of *Geotjeor*i soup were added to a mixer. Subsequently, *Geotjeori* was filtered using a filter paper, and the filtered liquid was measured using Digital Pocket Refractometer (PAL-1; Atago Co., Japan).

### 2.6 Analysis of texture (hardness)

After DBD plasma treatment, CT3 texture analyzer (Brookfield Engineering Laboratories Inc., Middleboro, MA, USA) was used for texture analysis. Hardness was measured after cutting *Geotjeori* to a certain size of 2.5 × 2.5 × 0.5 cm (L × W × T). Using probe TA39 (diameter 2 mm), the trigger load of the probe was set to 5.0 g and test speed to 5 mm/s. When a force of 5.0 g or more was applied to the sample, it was measured by cutting up to 70% of the sample deformation. It was used TPA mode, and all experiments were performed in triplicates.

### 2.7 Statistical analysis

Statistical analysis was conducted to identify significant differences between the experimental results. Data are presented as the mean of three replicates ± standard deviation. Statistical analysis was performed by applying one-way analysis of variance using the SPSS software program (version 12.0; SPSS Inc., Chicago, IL, USA). *Y. enterocolitica* and *S. aureus* represented as logarithmic functions, pH, Brix value, and Texture (hardness) were analyzed using Duncan's multiple range test. The D-values for the two bacteria were analyzed by applying *t*-test using the SPSS software. Statistical significance was set at the 5% level (*P* < 0.05).

## 3 Results

### 3.1 Effect of DBD plasma on reduction in Y. enterocolitica and *S. aureus* count in Geotjeori

In this study, *Geotjeori* was treated with DBD plasma for 5, 10, 20, 30, and 60 min to evaluate reduction in *Y. enterocolitica* and *S. aureus* populations. Both microbial populations in *Geotjeori* were gradually decreased after treatment with DBD plasma (*P* < 0.05) (Table 2). DBD plasma treatment for 5, 10, 20, and 30 min decreased *Y. enterocolitica* concentration by 0.09, 0.19, 0.34, and 0.64 log_10_ CFU/g, respectively (*P* < 0.05). In particular, it showed significant inactivation of *Y. enterocolitica* with a decrease 1.13 log_10_ CFU/g at 60 min. *Staphylococcus aureus* count was 0.09 log_10_ CFU/g at 5 min of DBD plasma treatment, which was not significantly different from the initial *S. aureus* count. However, it decreased significantly at 10, 20, and 30 min (*P* < 0.05) by 0.19, 0.45, and 0.72 log_10_ CFU/g, respectively. These results, confirmed that *Y. enterocolitica* and *S. aureus* count were decreased by > 1 log_10_ after treatment with DBD plasma for 60 min.

**Table 2:** Effects of dielectric barrier discharge (DBD) plasma treatment on reduction of *Yersinia enterocolitica* and *Staphylococcus aureus* count in *Geotjeori*.

Bacteria	DBD plasma treatment (min)	log_10_ CFU/g	log_10_ reduction
*Y. enterocolitica*	0	4.42 ± 0.10^a^	–
	5	4.30 ± 0.06^a^	0.12
	10	4.23 ± 0.10^ab^	0.19
	20	4.08 ± 0.11^b^	0.34
	30	3.78 ± 0.14^c^	0.64
	60	3.29 ± 0.14^d^	1.13
*S. aureus*	0	4.69 ± 0.05^A^	–
	5	4.60 ± 0.05^AB^	0.09
	10	4.50 ± 0.03^B^	0.19
	20	4.24 ± 0.03^C^	0.45
	30	3.97 ± 0.05^D^	0.72
	60	3.57 ± 0.12^E^	1.12

Values are represented as the mean ± standard deviation of three samples. Different letters (a–d for *Y. enterocolitica*, A–E for *S. aureus*) indicate significant differences (*P* < 0.05) in microbial count reduction according to Duncan's multiple range test at 5% probability.

**Fig. 2:**
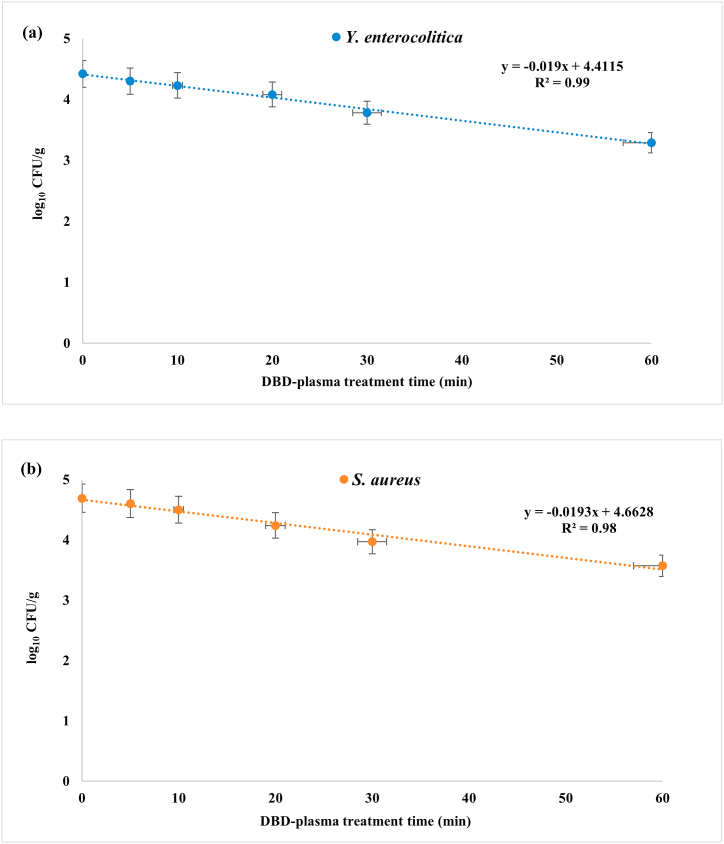
Fitted survival curves of *Y. enterocolitica* (a) and *S. aureus* (b) from dielectric barrier discharge (DBD) plasma treated *Geotjeori* using the first-order kinetic model (three samples/treatment).

Based on the *Y. enterocolitica* and *S. aureus* survival curves in *Geotjeori*, D-values (90% reduction) were calculated using the first-order kinetics (Table 3). The R^2^ values of 0.99 and 0.98 indicated that this log-linear kinetic model for *Y. enterocolitica* (Fig. 2a) and *S. aureus* (Fig. 2b) was a proper fit to determine slopes and D-values. No significant difference (*P* > 0.05) was observed in the D-values for the two bacteria in DBD plasma-treated *Geotjeori* (D = 52.83 and 51.95 for *Y. enterocolitica*, and *S. aureus*, respectively), indicating that the linear first-order kinetic model was appropriate for demonstrating *Y. enterocolitica* and *S. aureus* inactivation in *Geotjeori*.

**Table 3:** D-value for *Yersinia enterocolitica* and *Staphylococcus aureus* in *Geotjeori* treated with dielectric barrier discharge (DBD) plasma measured using the first-order kinetics model.

	*Y. enterocolitica*	*S. aureus*
Correlation coefficient (R^2^)	0.99	0.98
aD-value after DBD plasma treatment (min)	52.83 ± 2.94 ^NS^	51.95 ± 3.54

Values are represented as the mean ± standard deviation of three samples.

^NS^not significant values means within the same row (*P* > 0.05) according to *t*-test.

a D = −1/slope of a plot of log_10_ CFU/g versus time of DBD plasma treatment.

### 3.2 Effects of DBD plasma on the quality of Geotjeori

To investigate the effect of DBD plasma treatment on *Geotjeori*, pH, Brix value, and hardness as the main quality parameters were measured. Compared with untreated samples, those treated with DBD plasma (30 and 60 min) were not significantly different in terms of pH, Brix value, and hardness (*P* > 0.05) (Table 4).

**Table 4:** pH, Brix value, and hardness of *Geotjeori* after dielectric barrier discharge (DBD) plasma treatment.

Treatment duration (min)	pH	Brix value	Hardness (kg)
Control	6.03 ± 0.01 ^NS^	6.47 ± 0.06 ^NS^	2.06 ± 0.09 ^NS^
30	6.03 ± 0.01	6.37 ± 0.06	2.02 ± 0.02
60	6.02 ± 0.03	6.47 ± 0.06	2.08 ± 0.11

Values are expressed as the mean ± standard deviation of three samples.

^NS^ not significant values within the same column (*P* > 0.05) according to Duncan's multiple range test.

## 4 Discussion

In this study, *Y. enterocolitica* and *S. aureus* were inoculated into *Geotjeori* to investigate microbial inactivation by DBD plasma treatment. Agricultural products such as cabbage use kimchi may lose their natural flavor and texture, or nutrient destruction and discoloration may occur, so a non-chemical and non-thermal sterilization method is required [17]. In particular, unlike *kimchi*, *Geotjeori* is consumed immediately without fermentation, and this intake method is more likely to be microbiological contamination than fermented *kimchi*. This is because fermentation in food produces various organic acids such as acetic acid, which prevents pathogenic bacteria from growing well. In spite of these microbiological risks, no research has been reported on *Geotjeori*. In the case of *Geotjeori*, it is likely to be contaminated with *S. aureus* when seasonings are mixed with hands. In addition, it can also be stored in a refrigerator before consumption, it can be ontaminated with *Y. enterocolitica*, a psychrotrophic bacterium.

Currently, sterilization treatment technologies mainly used to maintain food quality and improve shelf life include heat, UV and chemical treatment. However, these sterilization technologies cause changes in the composition and quality of food due to high temperatures, or various problems arise due to the residual chemical components and production of harmful substances. Therefore, atmospheric pressure plasma was used as non-thermal sterilization technology that does not affect the quality of *Geotjeori* and has no risk due to chemical components. In addition, further research is needed on reduction of microbial contamination by pathogenic microorganisms using non-thermal plasma in food is still lacking.

The degree of inhibition of microorganisms varies depending on the type of injection gas used. Nitrogen, helium, or argon are generally used for antimicrobial studies of atmospheric pressure plasma. Son and Lee [18] reported complete sterilization against *E. coli* in a medium by DBD plasma using a mixture of argon and oxygen. Deng et al. [19] concluded that it is more effective to use atmospheric helium gas than an atmospheric mixture of helium and oxygen for inactivation of *Bacillus subtilis* spores. They reported that *B. subtilis* spores exposed to atmospheric pressure glow discharge plasma (3.5–6.5 kV, He) for 10 min were reduced to 1.8–4.0 log. Unlike the above studies, this study using nitrogen gas showed microbial sterilization effect (maximum reduction of 1.13 and 1.12 log_10_ CFU/g after DBD plasma treatment for 60 min against *Y. enterocolitica* and *S. aureus*, respectively) without adverse effects on the overall food quality. This finding was consistent with the study by Kim et al. [20], who reported that using nitrogen gas for DBD plasma can prevent oxidation and degradation of the sample by oxygen [20]. In addition, Sureshkumar et al. [21] reported that nitrogen plasma had a significant effect on sterilization due to the generation of ultraviolet (UV) radiation in a bacterial inactivation study using low-temperature plasma, and Hertwig et al. [22] also reported that N_2_-plasma obtained the highest inactivation efficiency for *B. subtilis* spores with significant intensity in the UV-C range than four other gasses-plasma (air, N_2_, O_2_, CO_2_).

The inactivation of *S. aureus* and *Y. enterocolitica* by non-thermal plasma could be associated with active species such as ROS, RNS and O_3_. These active species could directly or indirectly induce stress on the bacteria by mediating oxidation of lipids and proteins in cell membranes [23,24]. Since ROS such as oxygen atoms, ozone, peroxide, superoxide, hydroxyl radicals, and nitrogen species (N_2_, N_2_^+^) generated by plasma have strong oxidizability and interact easily with bacteria, they can oxidize the cell membrane and cause cytoplasm leakage [25]. The interaction of these active species with peptidoglycan and lipopolysaccharide results in damage to C–O, C–N, and C–C bonds, destroying the cellular molecular structure [24]. In addition, the electric and UV radiation generated during plasma treatment cause thymine dimer generation to damage DNA and inhibit bacteria [26]. It is judged that a suitable time for commercial application to completely inactivate microorganisms is about 1 h or more, and DBD plasma is cost-effective and commercially safe without producing hazards materials. Also, it can be used for large scale for inactivation of microorganisms. However, ROS and RNS produced in the surrounding air by the release of plasma can create toxic gases, including ozone, nitrogen dioxide (NO_2_), and NO. These gases are generally considered as air pollutants that have harmful effects on the human respiratory system. Mann et al. [27] reported that NO_2_ is produced by oxidation of NO, in the presence of oxygen or other oxidants. When the plasma treatment is performed in an atmospheric environment, NO_2_ is discharged in the surrounding treatment area; its odor threshold was set to 0.44 ppm or 0.9 mg/m^3^. According to the EU guideline 2008/50/EG, the daily limit of 40 mg/m^3^ is recommended, which has not been exceeded, suggesting no risk to human health owing to the generation of toxic gases during plasma treatment.

The processing power, exposure time, and exposure distance of DBD plasma are closely related to the degree of microbial inhibition, and the shorter the processing power and exposure time, the higher the microbial mortality rate [28]. In a study by Song et al. [17], DBD plasma treatment was performed on dry peppers to investigate the mortality rate of *S. aureus* according to the changes in treatment power (0.5–1.5 kW), exposure time (1–15 min), and exposure distance (10–35 mm). At the highest power (1.5 kW), 66.6% of microbial population was killed, and at the longest exposure time (15 min) and shortest exposure distance (10 mm), mortality rate was 82.6% and 71.4%, respectively. In a study by Kim et al. [29], *S. aureus* was cultured in a medium and DBD plasma treatment was performed to evaluate quantitative reduction due to death, which was observed to be 27% after treatment for 1 h. The results of the present study, show higher sterilization power than that of the Kim's study, with reduction in *S. aureus* count by 1.12 (approximately 92.41% reduction) after 1 h treatment. Greater effectiveness of DBD plasma treatment in the present study might be due to the difference in the exposure distance 30.4 cm versus 3 mm, Kim's study versus present study. Won et al. [30] reported that *E. coli* O157:H7, *Salmonella* enteritis, and *Listeria monocytogenes* in onion powder treated with DBD cold plasma at 9 kV for 20 min using helium as the plasma gas, showed a decrease by 1.4 ± 0.5, 2.3 ± 0.3, and 1.2 ± 0.0 log_10_ CFU/cm^2^, respectively. However, this study showed respective decrease of 0.34 and 0.45 log_10_ CFU/cm^2^ for *Y. enterocolitica* and *S. aureus* treated under 1.1 kV for 20 min, indicating that the sterilization effect in the study using onion powder was greater than in this study. This might be due to higher voltage despite the same processing time in the study using onion powder than in this study. In addition, Kim et al. [28] inoculated cabbage and lettuce with *Salmonella* typhimurium, treated them with microwave non-plasma at 900 W for 5 min, and observed microbial inhibition by 1.5 ± 0.2 and 1.1 ± 0.1 log_10_ CFU/cm^2^, respectively. *Y. enterocolitica* and *S. aureus* populations were decreased by 0.12 and 0.09 log_10_ CFU/cm^2^, respectively, at 5 min, revealing greater effect of higher sterilization power than in this study. Increase in the voltage used in the discharge plasma increases the frequency at which radicals are generated to prevent further activation of gas ionization, dissociation, and growth of food poisoning bacteria [31]. In addition, considering the morphological characteristics of food, the inactivation effect of food contaminated with bacteria does not necessarily appear the same.

The first-order kinetic model is generally used for estimating microbial inactivation, such as the D-value [32]; it provides useful information, including quantitative microbial risk assessment, and can compare significant differences in microbial reduction [16]. If the R^2^ value is higher than 0.90, it fits the first-order kinetic model. The R^2^ values for *Y. enterocolitica* and *S. aureus* were obviously higher than 0.90 in this study; therefore, we used the first-order kinetic model as the most suitable method for investigating inactivation of *Y. enterocolitica* and *S. aureus* using DBD plasma.

As DBD plasma treatment is used as an antimicrobial intervention for food, food quality should be considered for consumer acceptance. Therefore, in the current study, pH, Brix value, and hardness were measured to determine whether DBD plasma treatment affects the quality of *Geotjeori*. The pH of *Geotjeori* after DBD treatment was the same as the initial pH (5.0–6.0) of non-fermented *kimchi*. The overall quality of *Geotjeori* after DBD plasma treatment did not differ significantly. Lee et al. [33] measured the pH and hardness of brown rice after treatment with DBD plasma and observed only slight but no significant difference in pH from that of non-treated samples. However, they reported decrease in hardness owing to the absorption of moisture during cold plasma treatment. In contrast, Choi et al. [34] observed no significant difference in the texture of oyster after treatment with DBD plasma. Similar results were observed for hardness measurement of *Geotjeori* treated with DBD plasma in this study; it did not differ from that of untreated samples. Moisture loss from the surface of vegetables and fruits is common; however, only the extracellular fluid and not the cellular fluid may escape during this process. Further, a previous study has reported that less than 5% of moisture loss does not affect tissue hardness [35].

DBD plasma treatment exerts antimicrobial effect without significant changes in physicochemical quality parameters. However, in addition to these quality evaluations, additional studies, on nutritional and sensory analyses of *Geotjeori* treated with DBD plasma are needed. Several studies have reported the antimicrobial effect of DBD plasma, but studies on antimicrobial effect of DBD plasma against diverse spoilage and pathogenic microorganisms, including molds and viruses, in many fresh, semi-processed, and processed foods are still insufficient. Also, Butscher et al. [36] reported that the highest treatment conditions do not match the conditions required for high microbial reduction, and research to further enhance the antibacterial effect of plasma treatment while reducing the thermal effect should be conducted. In particular, since it is not easy to inactivation of bacteria that contaminate vegetables [37], it can be meaningful basic data for the agricultural industry. In order to commercially utilize DBD plasma technology, an accurate understanding of the mechanism is required, and additional research is needed to understand the interaction between various food components and DBD plasma. Therefore, this study provides basic data for antimicrobial effect of DBD plasma in *Geotjeori*.

## 5 Conclusions

The present study demonstrates the antimicrobial effect of DBD plasma against *Y. enterocolitica* and *S. aureus* in *Geotjeori*. DBD plasma treatment time for 5–60 min decreased *Y. enterocolitica* and *S. aureus* count by 0.12–1.13 and 0.09–1.12 log_10_ CFU/g, respectively. The D-value of DBD plasma calculated using the first-order kinetic model was 52.83 min (R^2^ = 0.99) for *Y. enterocolitica* and 51.95 min (R^2^ = 0.98) for *S. aureus*. DBD plasma treatment for 60 min was effective in reducing microbial count of both *Y. enterocolitica* and *S. aureus* by > 1 log_10_ CFU/g. There were no significant differences (*P* > 0.05) in pH, Brix value, and hardness between DBD plasma-treated and untreated samples of *Geotjeori*. These results suggest that DBD plasma is a potential physical non-thermal antimicrobial method that effectively decreased the microbial load from *Geotjeori* without altering its quality.

## Author contribution statement

So Hee Kim: Conceived and designed the experiments; Performed the experiments; Wrote the paper.

Sung-Hee Park: Analyzed and interpreted the data.

Sung Gi Min, Shin Young Park: Contributed reagents, materials, analysis tools or data

1) conceived and designed the experiments
2) performed the experiments
3) analyzed and interpreted the data
4) contributed reagents, materials, analysis tools or data
5) wrote the paper.

## Data availability statement

Data will be made available on request.

## Declaration of competing interest

The authors declare that they have no known competing financial interests or personal relationships that could have appeared to influence the work reported in this paper.
